# Switch From rhPTH1-84 to TransCon PTH With Individual Dose Adjustment in Adult Hypoparathyroidism—4-Week Results

**DOI:** 10.1210/jendso/bvaf113

**Published:** 2025-06-30

**Authors:** Heide Siggelkow, Kim A Peschke, Elena Tsourdi, Lorenz C Hofbauer, Christina M Berr, Stefanie Hahner, Christian Lottspeich, Ralf Schmidmaier, Martina Blaschke

**Affiliations:** Department of Trauma, Orthopedics and Reconstructive Surgery, University Medical Center Göttingen, Göttingen 37075, Germany; MVZ endokrinologikum Göttingen, Center for Endocrinology, Osteology, Rheumatology, Nulear Medicine and Human Genetics, Göttingen 37075, Germany; Department of Trauma, Orthopedics and Reconstructive Surgery, University Medical Center Göttingen, Göttingen 37075, Germany; Division of Endocrinology and Bone Diseases, Department of Medicine III, University Center for Healthy Aging, Technische Universität Dresden, Dresden 01307, Germany; Division of Endocrinology and Bone Diseases, Department of Medicine III, University Center for Healthy Aging, Technische Universität Dresden, Dresden 01307, Germany; Department of Endocrinology, I. Medical Clinic, University Hospital, University of Augsburg, Augsburg 86156, Germany; Department of Medicine I, Division of Endocrinology and Diabetes, University of Würzburg, Würzburg 97080, Germany; Department of Medicine IV, LMU University Hospital, LMU Munich 80336, Germany; Department of Medicine IV, LMU University Hospital, LMU Munich 80336, Germany; MVZ endokrinologikum Göttingen, Center for Endocrinology, Osteology, Rheumatology, Nulear Medicine and Human Genetics, Göttingen 37075, Germany; Clinic of Gastroenterology, Gastrointestinal Oncology and Endocrinology, University Medical Center Göttingen, Göttingen 37075, Germany

**Keywords:** palopegteriparatide, chronic hypoparathyroidism, treatment switch, parathyroid hormone, active vitamin D, hormone therapy

## Abstract

**Background:**

Replacement therapy with recombinant human PTH (rhPTH1-84) represents a causal treatment for patients with chronic hypoparathyroidism (HypoPT). Recently, palopegteriparatide (TransCon PTH), a novel long-acting drug with slow release of PTH1-34, was approved by the European Medicines Agency and Food and Drug Administration for treatment of HypoPT. To date, no data exist on the treatment switch from rhPTH1-84 to TransCon PTH.

**Methods:**

We retrospectively analyzed clinical data from 40 patients with chronic HypoPT during the switch from rhPTH1-84 to TransCon PTH. Independent of the last prior rhPTH1-84 dose, all patients were started on 18 µg of TransCon PTH as recommended by the manufacturer. TransCon PTH dose adjustments, changes in additional medication, and adverse events were documented during the treatment switch.

**Results:**

Within the first month after the treatment switch, 80% (n = 32) of patients needed individual adjustment of their TransCon PTH dose to achieve normocalcemia. Dose reduction (to 9-15 µg) was necessary in 38% (n = 15) and an increase (to 21-27 µg) in 43% (n = 17) of patients. Adjustments occurred predominantly (in 62% cases) according to serum calcium levels, partly dependent on symptoms. The prior applied rhPTH1-84 dose correlated significantly with the adjusted TransCon PTH dose (r = 0.4; *P* = .01). The treatment change was associated with moderate or mild adverse events in 24/40 patients.

**Conclusion:**

We hereby report the first clinical data on switching treatment from rhPTH1-84 to 18 µg TransCon PTH independent of the prior rhPTH1-84 dose. Our data support discrete adaptation of the starting dose depending on the prior rhPTH1-84 dosage.

Hypoparathyroidism (HypoPT) is characterized by the presence of hypocalcemia resulting from insufficient or absent secretion of PTH from the parathyroid glands [1]. Until recently, patients were only treated with calcium and active vitamin D3, often in combination with native vitamin D and magnesium [2-4]. However, there is growing evidence that either the disease itself or the use of conventional treatments is associated with a number of complications for these patients, including cataracts, intracerebral calcifications, renal dysfunction and kidney stones, cardiac arrhythmia and ischemic heart disease, depression, and increased mortality [5-9].

Historically, replacement of the missing hormone began with PTH1-34 predominantly in children and in some adults with refractory disease [10, 11]. Owing to its very short half-life, it required administration twice a day or via a pump. In 2018, rhPTH1-84, formerly used to treat osteoporosis [12], was repurposed and approved by the European Medicines Agency as NATPAR/NATPARA^®^ for adults with chronic hypoparathyroidism whose disease cannot be adequately controlled by standard therapy alone [3]. Since then, the medication was used to treat patients with HypoPT [13-15]. In October 2022, the manufacturer announced the global discontinuation of NATPAR/NATPARA production, due to unresolved supply issues. A new alternative treatment, TransCon PTH, was recently developed as a prodrug with sustained release of PTH1-34, ensuring stable PTH levels for 24 hours [16]. After it had been investigated in a phase 3 trial [17-19], it was approved by the European Medicines Agency as palopegteriparatide (Yorvipath^®^) in 2023 and by the Food and Drug Administration in 2024, for the treatment of adults with chronic HypoPT.

The phase 3 trial of TransCon PTH included solely HypoPT patients on conventional treatment [18]. Thus, there are currently no published data on switching patients from rhPTH1-84 to TransCon PTH. However, as a result of the pending termination of production of rhPTH1-84, the treatment switch of a significant number of patients currently on rhPTH1-84PTH is imminent. Here, we present our initial experience with switching patients from rhPTH1-84 to TransCon PTH in order to assist colleagues in the process of switching their own patients to TransCon PTH treatment in other parts of the world.

## Methods

### Study Design

The study presented is a retrospective, observational, multicenter cohort study including all patients on rhPTH1-84 treatment in the participating centers that were switched to TransCon PTH between August 2023 and September 2024. TransCon PTH was officially available from February 2024. The patients in Göttingen and Dresden were in part switched to TransCon PTH within a compassionate-use program starting in 2023.

The study was approved by the institutional review board of University Medical Center Göttingen (approval number:) (22/1/2025). Patients gave written consent for analysis and publication of their anonymized data. All procedures were performed locally in each of the centers (Augsburg, Dresden, Göttingen, Munich, and Würzburg) in accordance with the clinical requirements and available information on changes of dose, laboratory values, medication, and symptoms retrieved from electronic files and documentation. All data were summarized and analyzed in the center in Göttingen. The initial dose was suggested by the manufacturer on the basis of their experience in a few only personally reported cases.

### Study Participants

In Germany, patients with chronic HypoPT not adequately controlled through conventional therapy were eligible to be treated with rhPTH1-84 on a daily basis with the doses 25 µg, 50 µg, 75 µg, or 100 µg, in addition to the concurrent medication with calcium, active vitamin D3, native vitamin D, and magnesium. Inadequate control was defined as either required calcium levels that could not be reached with conventional treatment or the occurrence of side effects and complications. Owing to the fact that the maximum dose allowed was 100 µg rhPTH1-84, a number of patients also needed additional concurrent medication with active vitamin D; calcium, either regularly or on demand; native vitamin D; and/or magnesium to maintain their calcium serum levels in the target range. In some patients, rhPTH1-84 was temporarily increased to doses above 100 µg due to an intolerance to oral calcium and/or active vitamin D. In Germany, calcitriol and alfacalcidol are widely available and are prescribed according to personal preferences or patient tolerance. The last rhPTH1-84 dose was administered the day before starting 18 µg TransCon PTH. The 18-µg dose was recommended by the manufacturer (personal communication), and the 18-µg starting dose now represents the recommended dose for the approved drug when starting from conventional treatment. Active vitamin D medication remained unchanged, and additional calcium was provided on demand to relieve any symptoms of hypocalcemia. Laboratory monitoring varied from center to center, depending on local resources. Calcium levels were typically checked on days 4 and 7 and then weekly but were monitored more frequently in cases of hypo- or hypercalcemia. Quality of life was documented at baseline and thereafter with different questionnaires; however, these are not part of this analysis. Adverse events were recorded partly through structured documentation forms or from what patients reported at each visit.

### Statistical Analysis

All statistical analyses were performed with IBM-SPSS version 29 (IBM Corp., Armonk, NY, USA). The 2-sided Student's *t*-test was applied to compare 2 groups. The Kruskal-Wallis test for nonparametric distributed values was performed to compare more than 2 groups, and post hoc Dunn's multiple comparison test was performed. Pearson's test was used to correlate values (rhPTH1-84 and TransCon PTH) with normal distribution. A *P*-value <.05 was considered statistically significant. Graphics were generated using GraphPad Prism 10. We applied a calculation model to calculate the concurrent medication at baseline in comparison to the time point of 4 weeks. To identify the influence on calcium values, we created 6 groups of concurrent medication with increasing influence and appointed different scores to the groups. No medication was rated with 0 points, calcium on demand 1 point, calcium as daily regimen 2 points, active 1,25 D3 3 points, active D3 and calcium on demand 4 points, and active vitamin D and calcium as daily calcium 5 points. We analyzed the correlation of the concurrent medication at baseline with the TransCon PTH dose at 1 month.

## Results

We investigated 37 patients with postsurgical and 3 patients with hereditary HypoPT who had previously undergone treatment with rhPTH1-84 at doses of 25 µg, 50 µg, 75 µg, 100 µg, or >100 µg. These patients were switched to TransCon PTH between August 2023 and September 2024. Patients were recruited in Göttingen (n = 22), Munich (n = 8), Dresden (n = 4), Augsburg (n = 3), and Würzburg (n = 3). Patients were mainly female (n = 37; 93%), and their mean age was 54 ± 14 years. The average previous rhPTH1-84 dose was 64.7 ± 31 µg per day. Duration of prior treatment was 4.3 ± 1.7 years. Laboratory values at baseline are depicted in Table 1.

**Table 1.** Laboratory values at baseline in all patients

Serum values	Reference range	Mean ± SD (n) range
Calcium corr. mmol/L	2.15-2.5 mmol/L	2.17 ± 0.27 (40) 1.51-2.77
Phosphate mmol/L	0.8-1.6 mmol/L	1.22 ± 0.28 (40) 0.68-1.84
CPP mmol^2^/L^2^	<4.4 mmol^2^/L^2^	2.70 ± 0.57 (40) 1.29-3.79
Magnesium mmol/L	0.66-1.07 mmol/L	0.75 ± 0.10 (40) 0.49-0.92
GFR mL/min	>60 mL/min	77.75 ± 17.90 (40) 38-114

Abbreviations: corr., corrected; CPP, calcium-phosphate product; GFR, glomerular filtration rate.

As presented in Table 2, 93% (n = 37) of patients were also taking native vitamin D supplements as a concurrent medication, 20% (n = 8) calcitriol, and 12% (n = 5) alfacalcidol as active vitamin D medication. Calcium was part of the daily regimen for 32% of patients (n = 13), while 57% (n = 23) used it only as needed on demand. Magnesium was included in the standard treatment regimen for 60% of patients (n = 24).

**Table 2.** Medication at baseline with rhPTH-1-84 and after 1 month on TransCon PTH

	Baseline		After 1 month	
	Medication n (%)	Independent from n (%)	Medication n (%)	Independent of n (%)
PTH medication	40 (100)		40 (100)	
rhPTH1-84				0 (0)
TransCon PTH		0 (0)		
Comedication	40 (100)		40 (100)	
Native vitamin D	37 (93)	3 (7)	37 (93**)**	3 (7)
Calcitriol	8 (20)	27 (68)	7 (17)	31 (78)
Alfacalcidol	5 (12)		2 (5)	
Calcium On demand	13 (32) 23 (58)	4 (10)	8 (20) 18 (45)	14 (35)
Magnesium On demand	24 (60) 4 (10)	12 (30)	24 (60) 4 (10)	12 (30)

Indicated are also the numbers and percentages of patients independent of the corresponding medication.

In addition, Table 3 presents the medication doses at baseline with rhPTH-1-84 and after 1 month on TransCon PTH. The comparison reflects a decrease in concurrent medication doses for native vitamin D and active vitamin D (calcitriol or alfacalcidol), as well as for calcium and magnesium as part of the regimen. Patients taking calcium or magnesium on demand were excluded due to variable doses per day.

**Table 3.** Medication (doses) at baseline with rhPTH-1-84 and after 1 month on TransCon PTH

	Baseline (n*) Mean ± SD	Baseline (n^+^) Mean ± SD	After 1 month (n*) Mean ± SD	After 1 month (n^+^) Mean ± SD
PTH medication µg/day	rhPTH1-84 64.7 ± 31.0 (40)		TransCon 18.5 ± 4.6 (40)	
Comedication				
Native vitamin D, IU/day	2309 ± 1158 (37)	2186 ± 1224 (40)	2271 ± 1214 (37)	2101 ± 1314 (40)
Calcitriol, µg/day	0.34 ± 0.13 (8)	0.08 ± 0.16 (40)	0.36 ± 0.13 (7)	0.06 ± 0.15 (40)
Alfacalcidol, µg/day	0.95 ± 0.91 (5)	0.12 ± 0.43 (40)	1.75 ± 1.10 (2)	0.09 ± 0.42 (40)
Calcium, mg/day	617 ± 332 (13)	472 ± 394 (17)	702 ± 389 (8)	255 ± 412 (22)
Calcium, on demand	Variable (23)		Variable (18)	
Magnesium, mg/day	484 ± 348 (24)	323 ± 365 (36)	468 ± 347 (24)	312 ± 359 (36)
Magnesium, on demand	Variable (4)		Variable (4)	

Mean values are calculated for patients taking the concurrent medication (at baseline and after 1 month, left column, N*) and are also calculated for all patients, except for patients taking the medication only on demand (at baseline and after 1 month, right column, N^+^).

^*^Mean values in patients taking the concurrent medication.

^+^Mean values in all patients with or without comedication as part of the daily regimen.

**Figure 1.**
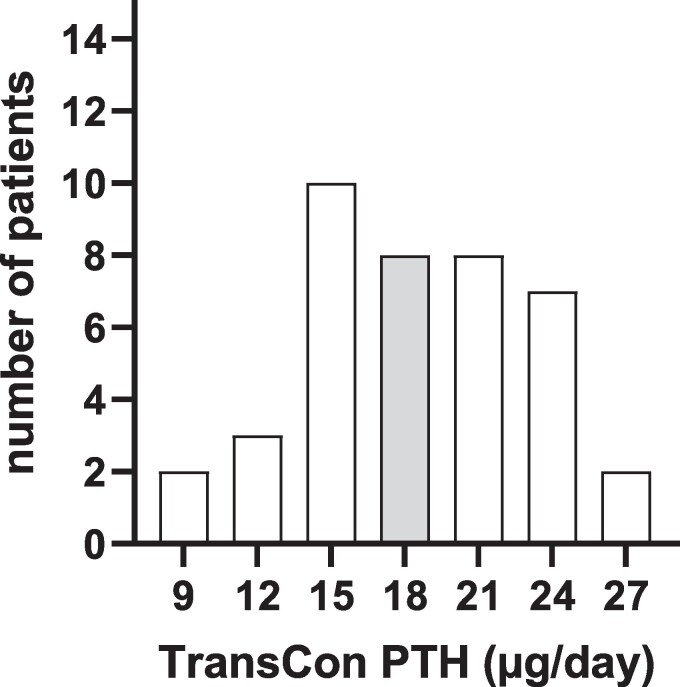
TransCon PTH dose 1 month after switch. The initial TransCon PTH dose is marked in gray.

At the time point of switching to TransCon PTH, all patients initially received the manufacturer's recommended dose of 18 µg TransCon PTH /day. During the first month, doses were adjusted weekly in 3 µg steps for each patient resulting in a mean dose of 18.5 ± 4.6 µg/day after 1 month. Comedication with active vitamin D could be stopped in 4 patients and daily calcium or calcium on demand in 10 patients after 1 month of taking TransCon PTH (Table 2). The number of patients and the individual doses of TransCon PTH after 1 month are presented in Fig. 1.

**Figure 2.**
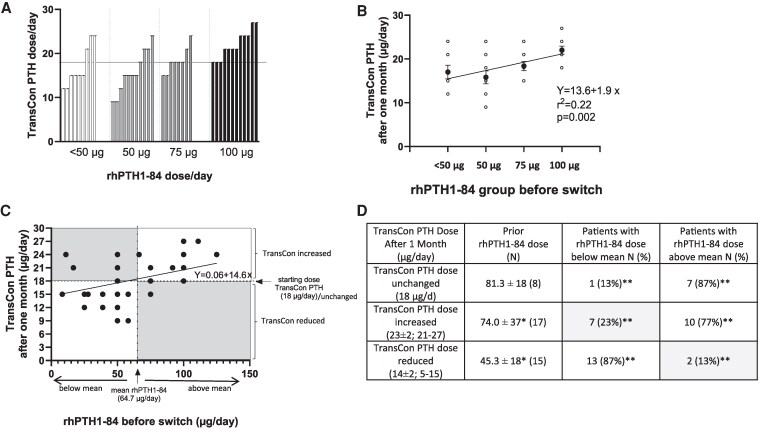
Changes in TransCon PTH dose after 1 month in relation to rhPTH1-84 dose. (A) Changes in TransCon PTH corresponding to rhPTH1-84 doses before the switch. (B) Linear regression of TransCon PTH according to the former rhPTH1-84 dose. Patients on the lower doses of rhPTH1-84 needed 3 µg/day lower doses of TransCon PTH after 1 month compared to those with doses above 75 µg. (C) Linear regression of TransCon PTH and rhPTH1-84 dose. The lower left white area represents patients with low rhPTH1-84 and reduced TransCon PTH doses. The upper right white area represents patients with high rhPTH1-84 and increased TransCon PTH doses. Patients within gray areas represent unexpected dose changes, mainly arising from complaints or corresponding calcium levels (D) The table depicts TransConPTH dose changes (increased or decreased TransCon PTH) as well as the number of patients after 1 month who were previously treated with rhPTH1-84 doses above and below the mean [gray cells correspond to gray cells in (C)]. *P < .01 2-sided *t*-test; **P < .0001 Fisher's exact test.

Furthermore, we were interested in whether the TransCon PTH dose after 1 month correlated with the previously applied rhPTH1-84 dose. As demonstrated in Fig. 2, most patients (73%; 9 out of 12) needed an increase in the dose of TransCon PTH if the final dose of rhPTH1-84 was 100 µg rhPTH1-84 (or higher; Fig. 2A). If the final rhPTH1-84 dose was in the lower range (50 µg or below), the TransCon PTH dose needed to be reduced in 65% of patients (13 out of 20). In 50% of the cases in whom the final dose of rhPTH1-84 was 75 µg (4 out of 8), no adjustment to the TransCon PTH dose was necessary (Fig. 2A). As demonstrated in Fig. 2C, patients were assigned to 2 groups, namely patients who were treated with rhPTH1-84 doses above the mean value (64.7 µg rhPTH1-84/day) and patients who were treated with rhPTH1-84 doses below the mean. As presented in Fig. 2D, patients needing an increased TransCon PTH dose within the first month (n = 17) were previously treated with higher rhPTH1-84 doses (*P* = .01; 2-sided *t*-test) before the switch, as compared to patients needing a reduced TransCon PTH dose (n = 15) (Fig. 2D). The respective percentages of patients needing an increase, a reduction, or no change according to the mean TransCon PTH dose were significantly different (Fisher's exact test *P* < .0001) (Fig. 2D).

The final doses of rhPTH1-84 and TransCon PTH correlated positively (r = 0.4; *P* = .01). By applying a linear regression (Fig. 2B and 2C), a significant but weak linear relationship between rhPTH1-84 and TransCon PTH doses can be demonstrated (r^2^ = 0.16, *P* = .01 and r^2^ = 0.22, *P* < .01), indicating that a 25-µg higher dose of rhPTH1-84 prior to transition would end in a 1.5-µg higher dose of TransCon PTH after 1 month of treatment in 16% of patients (r^2^ = 0.16). Age, sex, body mass index, length of treatment on rhPTH1-84, baseline calcium values, kidney function according to glomerular filtration rate, duration of HypoPT, or concurrent medication in multiple regression analysis all had no influence on the final dose of TransCon PTH.

**Figure 3.**
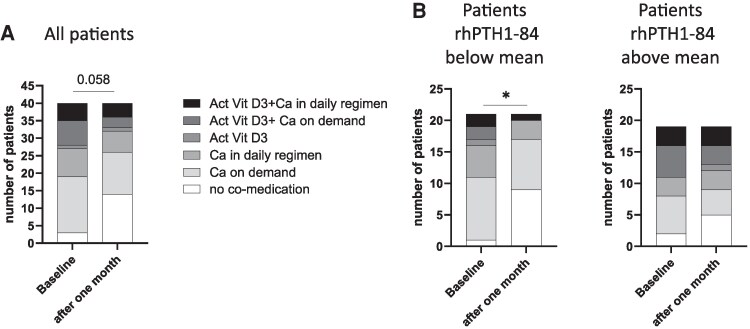
Changes in concurrent medication after 1 month in HypoPT patients after switching from rhPTH1-84 to TransCon PTH. (A) all patients (n = 40). (B) Patients separated by rhPTH1-84 dosage prior switch: patient group below rhPTH1-84 mean (64.7 µg/day; n = 21) or patient group above rhPTH1-84 mean (n = 19). * < .05 Fisher's exact; *P* = .058 Fisher's exact. Abbreviations: Act Vit D3, active vitamin D3; comedication, concurrent medication.

The concurrent medication at baseline did not correlate to rhPTH1-84 doses but was numerically different in those with lower rhPTH1-84 doses at baseline with less active vitamin D medication (24% vs 37%, *P* = .058). There was significantly less concurrent medication after 4 weeks in those patients treated with prior rhPTH1-84 below the mean. In total, in 17 patients (43%) the concurrent medication was decreased, in 21 patients the concurrent medication did not change, and in 2 patients the medication was increased (Fig. 3).

The relationship between concurrent medication in those with hypocalcemia, hypercalcemia, and normocalcemia is demonstrated in Table 4. Ten patients with higher rhPTH1-84 doses at baseline (82.8 ± 33 µg/day) had hypocalcemia; 14 patients with lower doses at baseline presented with calcium values indicating hypercalcemia and lower TransCon PTH doses after 1 month. Normocalcemia was seen in 15 patients with mean doses of 64 µg rhPTH1-84 at baseline (Table 4). The high percentage of patients with hypercalcemia was very surprising; in the analysis of concurrent medication, 57% of these patients were taking calcium on demand. Of patients with hypocalcemia during the first 4 weeks of treatment, 30% were treated with calcium on demand and active vitamin D3 and 30% with only calcium on demand as concurrent treatment (Table 4).

**Table 4.** rhPTH-1-84 and concurrent medication at baseline in patients classified by calcium values into normocalcemia, hypocalcemia, and hypercalcemia within the first 4 weeks after the switch to TransCon PTH

	Hypocalcemia < 2.15 mmol/L	Normocalcemia 2.15-2.5 mmol/L	Hypercalcemia > 2.5 mmol/L
Number of patients*^a^* (%)	10 (25)	15 (38)	14 (35)
Calcium min-max week 1 to 4 (mmol/L)	(1.8-2.1)	(2.2-2.49)	(2.53-3.1)
rhPTH1-84 baseline (µg)	82.8 ± 33	64.1 ± 30	50 ± 24
Transcon dose (µg) after 1 month	22.2 ± 4	19.8 ± 3	14.4 ± 4
Concurrent medication at baseline in patients with different calcium levels (%)			
Only native vitamin D	1 (10)	2 (13)	0
Calcium on demand	3 (30)	4 (27)	8 (57)
Calcium in regimen	2 (20)	3 (20)	3 (21)
Only Calcitriol/alfacalcidol	0	1 (7)	0
Calcium on demand and Calcitriol/alfacalcidol	3 (30)	2 (13)	2 (14)
Calcium in regimen and Calcitriol/alfacalcidol	1 (10)	3 (20)	1 (7)

^*a*^One patient with both hypo- and hypercalcemia during the first 4 weeks was excluded from this analysis.

**Figure 4.**
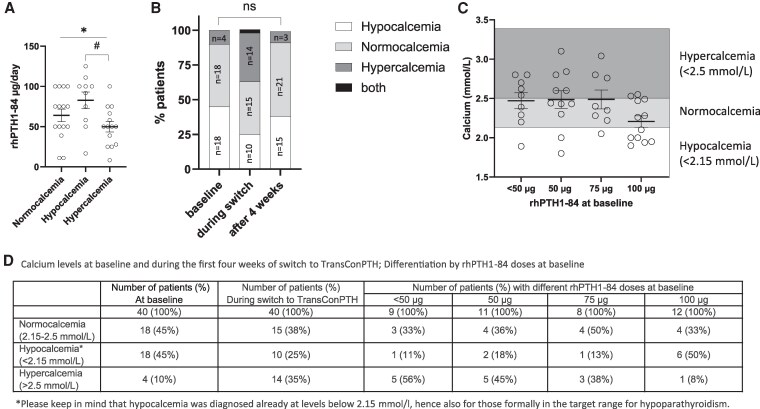
rhPTH1-84 daily dosage in patients separated into groups normocalcemia (2.15-2.5 mmol/L), hypocalcemia, and hypercalcemia. (A) Calcium levels during the first 4 weeks in relation to rhPTH doses at baseline *P < .05 Kruskal-Wallis; #Dunn's post hoc multiple comparison test. (B) Number of patients at baseline, during switch, and after 1 month displaying calcium levels (C) Calcium levels during switch to TransCon PTH distinguishing between different rhPTH1-84 doses at baseline. (D) Number of patients at baseline and during the first 4 weeks displaying calcium levels and rhPTH doses at baseline.

We performed further analysis to evaluate whether higher doses of rhPTH1-84 at baseline correlated to normocalcemia. Serum calcium values at baseline or after 4 weeks did not correlate with the dose of rhPTH1-84. However, the Kruskal-Wallis test for rhPTH1-84 dosage for normocalcemia, hypocalcemia, and hypercalcemia was significant with *P* = .034, due to the differences in hypo- and hypercalcemia (post hoc *P* = .025, Fig. 4A). In other words, normocalcemia was achieved more often in patients treated with 50 to 75 µg rhPTH1-84 (mean 64.1 ± 30, range 11-100 µg/day). Patients with higher (mean 82.8 ± 33; range 16.7-125) or lower rhPTH1-84 doses (mean 50 ± 24; range 8.3-100 µg/day) ended up more often with hypo- or hypercalcemia (Fig. 4).

Calcium values remained within normal limits (2.15-2.5 mmol/L) in the 1-month period in 15 patients (38%, Fig. 4B). Although calcium was within the normal range, TransCon PTH doses were still adjusted in some cases. Either the dose was increased (n = 47%; n = 7) or reduced (13%; n = 2) depending on symptoms or the need of additional calcium on demand. High serum calcium values (>2.5 mmol/L) were measured in 13 patients. This was mainly the case in patients treated with prior rhPTH1-84 doses lower than 60 µg/day rhPTH1-84 (77%; n = 10). Hypercalcemia was followed by a reduction in TransCon PTH dose, a reduction of active vitamin D, or a decrease in supplemental calcium or calcium on demand. Hypocalcemia (<2.15 mmol/L, n = 10) was mainly observed in patients with high prior rhPTH1-84 doses (67% had doses higher than 90 µg rhPTH1-84 prior to transition). TransCon PTH dose was increased in these patients. The mean doses in the groups normocalcemia, hypocalcemia, and hypercalcemia are depicted in Table 4.

The reasons for adjusting the dose were the continuous switch with a decrease in conventional treatment to increasing doses of TransCon PTH, serum calcium values outside the normal range (2.15-2.5 mmol/L) (n = 25; 62%), or adverse events resulting from the switch to TransCon PTH. Symptoms leading to dose changes were either headache followed by a temporary dose decrease or numbness or tingling despite normocalcemia, resulting in an increase of TransCon PTH.

We evaluated the data on the basis of the doses of rhPTH1-84 administered at 25, 50, 75, and 100 µg, respectively. The data demonstrate that 56% on doses lower than 50 µg and 45% on doses of 50 µg developed hypercalcemia during the first 4 weeks. Furthermore, 50% of patients on doses of 100 µg presented with hypocalcemia during the first month (Fig. 4C). Serum calcium levels remained within normal limits in 38% of patients during the first month. Hypercalcemia developed in 32%, and hypocalcemia in 25%.

**Figure 5.**
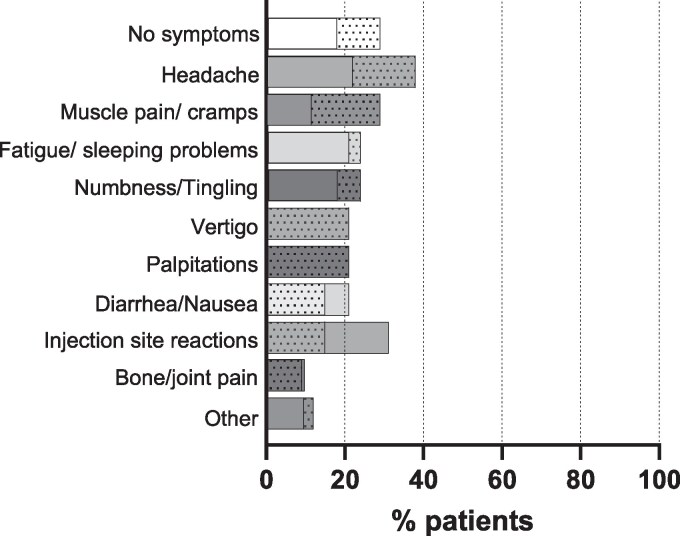
Documented or reported adverse events 1 month after treatment switch to TransCon PTH (n = 34 patients, dotted bars). The corresponding adverse events in the phase 3 trial with TransCon PTH after switching from conventional treatment [18] are also depicted (clear bars). Vertigo and palpitations were not documented separately in the phase 3 trial; therefore no clear bars are depicted for both symptoms.

During the treatment switch, patients were asked to document any new symptoms or adverse events. In 34 patients, such symptoms were documented (Fig. 5). Signs of hypocalcemia such as numbness and tingling were reported by 24% (n = 8) and injection-site reactions by 15% (n = 5). Among the 34 patients, 38% (n = 13) reported headache, 29% (n = 10) muscular spasms, 9% (n = 3) arthralgia, 21% (n = 7) vertigo, 15% other gastrointestinal symptoms (n = 5), 21% (n = 7) palpitations, and 24% (n = 8) sleep disturbance or fatigue. In 12% (n = 4) sweating, hypertension, increased blood pressure, hair loss, trembling, or visual changes were part of the complaints. Among the 34 patients, 29% (n = 10) did not report any adverse events. We compared the different parameters in those with and without symptoms and found no difference in the dose of thPTH1-84, the concurrent medication with calcium or calcitriol/alfacalcidol, or serum calcium values.

One patient who reported side effects including vertigo, palpitations, nausea, and local reaction at the injection site was seen by the attending physician. Treatment with TransCon PTH was subsequently paused for 4 days and restarted thereafter, resulting in relief of the symptoms. Patients also reported difficulties differentiating adverse events from long-standing symptoms of HypoPT.

## Discussion

In this study, we report on the first 40 patients successfully switched from rhPTH1-84 to TransCon PTH in Germany. Overall, the transition worked well with stopping the rhPTH1-84 treatment the day before and starting with 18 µg TransCon PTH daily. No emergency situations or hospital admissions arose; only 1 patient had to be seen in between scheduled visits to adapt to the medication regimen. No dose adjustment was needed during the 4 weeks in one-fifth of patients, whereas 38% needed an increase and 42% a decrease of the dose. There was a significant but weak correlation between the last applied rhPTH1-84 dose and the TransCon PTH dose after 4 weeks. We therefore further analyzed the data to evaluate for additional factors influencing the dose adjustment. When starting those with lower doses of rhPTH1-84, hypercalcemia occurred more often, and in those above 100 µg at start, hypocalcemia was present in 50% of patients. We therefore suggest that those with prior doses below 50 µg rhPTH1-84 start with 15 µg TransCon PTH, those with 50 or 75 µg rhPTH1-84 stay with 18 µg TransCon PTH at start, and those with prior doses above 75 µg rhPTH before the switch start with 21 µg TransCon PTH. Calcium control stays essential 7 days after the dose change for all patients.

Nevertheless, there were a number of exceptions. Six patients on rhPTH1-84 doses of 50 µg or lower needed TransCon PTH doses of 21 or 24 µg, and 2 patients on a prior dose of 75 µg rhPTH1-84 needed 15 µg TransCon PTH. Thus, the exact dose of TransCon PTH cannot be predicted in all patients based on the previous rhPTH1-84 dose, also due to the individual concurrent medication. However, our data may give some orientation regarding the range of doses after 4 weeks.

There were a number of symptoms reported by the patients, predominantly headache; vertigo; fatigue; palpitations; and pain in muscles, joints, and bone. Dependent on the calcium level, symptoms of hypocalcemia occurred, such as numbness, tingling, and muscle spasms, partly lasting the entire first 4 weeks. However, 10 of the 40 patients did not have any symptoms at all. Some of the symptoms known to occur in patients with hypoparathyroidism [20-22] correlated with serum calcium levels during transition and diminished on taking calcium orally on demand.

To date, no other data exist on the transition from rh PTH1-84 to TransCon PTH. In phase 2 and 3 trials of TransCon PTH [17, 19], participating patients were switched from conventional treatment to TransCon PTH; thus the situation for the patients was completely different. Hypocalcemia was reported in 6% in the phase 3 trial [19]; 10% of our patients presented with hypocalcemia, mainly those who were transferred from 100 µg rhPTH1-84. At baseline, some patients were below the normal range for calcium; however, the percentage did not differ from that after the switch. Nevertheless, headache was the most common symptom also in the phase 3 trial with 21%, followed by nausea, fatigue, hypertension, muscular spasm, and arthralgia [18]. Local site reaction was reported by 1 in 20 patients. The systemic effects, like headache and nausea, have also been described in other studies with PTH analogs used in osteoporosis treatment, such as teriparatide or abaloparatide, and are thought to be a class effect [23]. The suggestion is that these symptoms are caused by a systemic vasodilation effect that recedes after a few days [24, 25]. In our patients, the symptoms were generally moderate or mild, and medication with TransCon PTH was only transiently decreased in 1 patient to reduce side effects. In patients treated with TransCon PTH in the phase 3 trial, significant improvements in quality of life were reported [18], proving to be a source of motivation for all the patients in our study to continue treatment. Interestingly, the ability to use the medication at room temperature with no need for cooling after starting with the pen device was mentioned by a number of patients as a decisive advantage in comparison with the application of rhPTH1-84.

There are some limitations to our study. It was designed as a preplanned retrospective multicenter study documenting the regular change in treatment and not as a prospective study. Furthermore, the number of patients is small; the analysis of subgroups has to be viewed with caution. The duration of the study was very short, and the final dose of TransCon PTH was not reached during the first month of treatment. When reporting adverse events during transition, some patients may have confused their disease symptoms with side effects of the switch. As most patients were still in the transition period, results will be more informative after a longer period of treatment. However, the advantages of this design are the results from a real-world evidence study with different patients' subgroups. This helps to clarify how safe the treatment is for patients with different forms and severities of HypoPT. We would like to point out that keeping the active vitamin D and calcium dose unchanged should only be considered when switching from another PTH treatment to TransCon PTH. The switch from conventional treatment is different and was not investigated or discussed in this study.

In summary, we report on the first 40 patients undergoing a treatment switch from rhPTH1-84 to TransCon PTH worldwide. In general, the switch was without severe complications and with only mild or moderate side effects. Four weeks was too short a time to document the final dose and the full effect of the treatment switch; therefore, data on treatment dose, laboratory values, and quality of life several months after treatment switch will be of further interest. Our data facilitate a dose adaptation in patients switched from rhPTH1-84 to TransCon PTH. With our data, we hope to provide some assistance to colleagues in other countries, in which TransCon PTH has also received approval from the respective authorities and will be available to treat their hypoparathyroidism patients.

## Data Availability

The datasets used and/or analyzed during the current study are available from the corresponding author on reasonable request.
